# Ketogenic diet therapy for high-grade gliomas combined with standard treatment using an angiogenesis inhibitor: An exploratory pilot study on feasibility

**DOI:** 10.1093/noajnl/vdaf264

**Published:** 2025-12-22

**Authors:** Takashi Sasayama, Kazuhiro Tanaka, Hiroaki Nagashima, Michiko Takahashi, Misa Yamanishi, Misaki Kuroda, Satoko Tabuchi, Keisuke Hagihara, Shunsuke Yamanishi, Yusuke Ikeuchi, Yuichi Fujita, Hirohumi Iwahashi, Sayaka Hotta, Michiko Matsunaga, Shoji Sanada, Yoshihiro Muragaki, Masamitsu Nishihara

**Affiliations:** Department of Neurosurgery, Kobe University Graduate School of Medicine, Kobe; Department of Neurosurgery, Kobe University Graduate School of Medicine, Kobe; Department of Neurosurgery, Kobe University Graduate School of Medicine, Kobe; Department of Nutrition, Kobe University Hospital, Kobe; Department of Internal Medicine, Division of Diabetes and Endocrinology, Kobe University Graduate School of Medicine, Kobe (M.T.); Department of Nutrition, Kobe University Hospital, Kobe; Department of Nutrition, Kobe University Hospital, Kobe; Department of Nutrition, Kobe University Hospital, Kobe; Research Institute for Microbial Diseases (RIMD), Osaka ­University, Suita; Department of Neurosurgery, Kobe University Graduate School of Medicine, Kobe; Department of Neurosurgery, Kobe University Graduate School of Medicine, Kobe; Department of Neurosurgery, Kobe University Graduate School of Medicine, Kobe; Department of Neurosurgery, Kobe University Graduate School of Medicine, Kobe; Department of Respiratory Medicine and Clinical Immunology, Osaka University Graduate School of Medicine, Suita; Graduate School of Education, Kyoto University, Kyoto; Clinical and Translational Research Center, Kobe University Hospital, Kobe; Department of Medical Device Engineering, Kobe University Graduate School of Medicine, Kobe (Y.M.); Department of Neurosurgery, Nishi-Kobe Medical Center, Kobe

**Keywords:** angiogenesis inhibitor, feasibility, ketogenic diet, malignant glioma, safety

## Abstract

**Background:**

Altered tumor metabolism has renewed interest in ketogenic strategies, despite limited clinical evidence in glioma. Whereas the ketogenic diet (KD) alone elevates intratumoral amino acids, bevacizumab (BEV) co-administration suppresses these metabolites and curtails tumor growth, pointing to a synergistic therapeutic potential.

**Methods:**

We conducted a clinical pilot study to evaluate the combination of KD and standard therapy, combining BEV, in patients with malignant glioma. A standardized modified ketogenic diet (mKD) regimen was implemented: carbohydrate intake was restricted to 10 g/day in the first week, 20 g/day in the following 2 months, and ≤30 g/day thereafter. MCT oil was administered at ≥50 mL/day, and ketone formula supplements were provided as needed. The primary endpoint was to assess safety and feasibility.

**Results:**

10 patients were enrolled. The duration of mKD ranged from 63 to 1,954 days, with a median of 185 days. All patients showed a rapid increase in serum ketone levels and achieved therapeutically adequate glucose-ketone index values. All participants met the predefined safety criteria, and no severe adverse events were reported. One patient discontinued the diet owing to moderate abdominal pain. The objective response rate was 50%, and notably, one patient remained on mKD for more than 5 years without tumor recurrence. The median progression-free survival from mKD initiation was 9.5 months, and the median overall survival was 31 months.

**Conclusions:**

The combination of mKD and standard therapy with BEV was safe and feasible in patients with malignant glioma. Larger clinical trials are needed to determine its anti-tumor efficacy and clinical benefit.

Key Points

- In patients with malignant gliomas, the introduction of a modified ketogenic diet in combination with standard therapy, including bevacizumab, rapidly induced effective ketosis, was well tolerated, and demonstrated feasibility.

Importance of the Study

Alterations in tumor metabolism have led to increasing interest in dietary interventions such as the ketogenic diet (KD). While KD has demonstrated potential benefits in preclinical models, clinical evidence for its efficacy in glioma patients is still scarce. We conducted a clinical pilot study to evaluate the safety and feasibility of a modified ketogenic diet (mKD) in combination with standard therapy, including bevacizumab, for patients with malignant glioma. The dietary regimen restricted carbohydrate intake to 10∼30 g/day. All patients achieved rapid ketosis with therapeutically adequate glucose-ketone index (GKI) values, and no severe adverse events occurred. The median duration of mKD was 6.2 months. These findings indicate that mKD with bevacizumab is safe and feasible, warranting larger trials to clarify therapeutic efficacy.

Treatment of malignant glioma comprises multimodal therapy, including surgery, radiotherapy (RT), and chemotherapy. Surgery aims to remove as much of the lesion as possible; however, complete resection is challenging owing to tumor invasion and the need to preserve neurological function.^1^ The standard postoperative therapy for malignant glioma is RT combined with temozolomide (TMZ), followed by post-irradiation maintenance TMZ monotherapy.^2^ Internationally, RT + TMZ remains the standard of care, as this approach significantly improves survival. Two large double-blind studies investigated the addition of the anti-angiogenic agent bevacizumab (BEV) to the standard radiochemotherapy for primary glioblastoma, but neither showed a statistically significant improvement in overall survival (OS), which remained at approximately 16 months. In Japan, BEV is approved for newly diagnosed glioblastoma but is most often administered at recurrence. Given the extremely poor prognosis of malignant glioma, particularly glioblastoma, new therapeutic strategies are urgently needed.

When glucose is depleted during fasting, ketone bodies are produced in the liver to provide an alternative energy source for the brain.^3^ Acetoacetic acid and β-hydroxybutyric acid, both water-soluble, enter the bloodstream, are taken up by peripheral tissues including the brain, converted to acetyl-CoA, and subsequently enter the TCA cycle to generate ATP.^4^ Thus, ketone bodies are physiologically produced during fasting as an alternative fuel when glucose availability is limited. Fatty acids cannot cross the blood-brain barrier; hence, ketone bodies serve as the sole alternative to glucose for cerebral energy metabolism.^5^

Previously, we investigated the expression of ketone body–metabolizing enzymes, including 3-hydroxybutyrate dehydrogenase (BDH1) and succinyl-CoA:3-oxoacid CoA transferase (SCOT), in surgically resected malignant glioma specimens. These enzymes were markedly downregulated, indicating a limited capacity of glioma cells to convert ketone bodies into acetyl-CoA and thereby rendering them potentially more vulnerable to the metabolic constraints imposed by a ketogenic diet (KD).^6^ In a murine brain tumor xenograft model, KD alone led to an increase in several intratumoral amino acids, reflecting enhanced amino acid metabolism, whereas the addition of an angiogenesis inhibitor significantly reduced these metabolites. Moreover, the combination of KD with an angiogenesis inhibitor markedly prolonged survival in glioma-bearing mice.^6^ These findings suggest that the therapeutic efficacy of KD alone may be limited, whereas its combination with anti-angiogenic therapy may exert synergistic antitumor effects.

To date, several reports have described the use of KD in patients with malignant brain tumors.^7–14^ The first study, by Nebeling et al. in 1995, reported that patients with recurrent malignant glioma treated with KD survived for 4-5 years after diagnosis.^7^ Most recently, Amaral et al. conducted a phase I clinical study assessing the safety and feasibility of KD combined with standard of care in patients with newly diagnosed glioblastoma.^14^ Hagihara et al. studied 55 patients with stage IV cancer using a uniquely developed modified KD regimen; among them, 10 patients demonstrated a partial response or greater, with a median OS of 32.2 months, supporting the potential benefit of KD therapy.^15^ However, only one patient in that study had a brain tumor, and the efficacy and safety of KD specifically for malignant gliomas remain unestablished.

Existing studies on KD therapy for gliomas show considerable variation in treatment duration and study design; reported implementation periods ranged from 3 to 16 months. Owing to the small sample sizes and absence of statistical analyses, the anti-tumor effects of KD remain uncertain. Therefore, we evaluated the safety and feasibility of implementing KD therapy in combination with standard care with the anti-angiogenic drug BEV in patients with malignant glioma.

## Methods

### Ethical Considerations

This study was conducted between July 2018 and March 2025 and was registered with UMIN Clinical Trials Registry (UMIN000031308) on April 1, 2018. The protocol was approved by the institutional review board of Kobe University Hospital (No. 300001), and all patients provided written informed consent before enrollment. All procedures were carried out in accordance with relevant guidelines and regulations.

### Study Design and Objectives

This single-institution pilot study was conducted at Kobe University Hospital in accordance with International Conference on Harmonisation guidelines. The primary objective was to evaluate the safety and feasibility of implementing modified ketogenic diet (mKD) therapy in combination with standard treatment with the anti-angiogenic agent BEV, in patients with newly diagnosed or recurrent malignant glioma. The duration of mKD therapy was recorded, and feasibility was assessed based on the proportion of patients who continued the mKD for 3 and 6 months, with feasibility considered acceptable if at least half of the patients were able to continue. Adverse events were monitored according to the Common Terminology Criteria for Adverse Events Version 5.0 guidelines. The secondary objectives were to assess the 1-year survival rate, OS time, PFS, tumor response rate, changes in blood ketone body levels during mKD therapy, and the incidence of adverse events. This study was designed as a translational step toward future clinical trials evaluating the efficacy of this combined therapeutic approach.

### Inclusion and Exclusion Criteria

Patients aged over 20 years with newly diagnosed or recurrent malignant glioma were eligible for this study. Malignant glioma included glioblastoma, grade 3-4 astrocytoma, grade 3 oligodendroglioma, diffuse midline glioma (DMG), high-grade glioma (not otherwise specified (NOS)), and all types of recurrent gliomas. Eligible patients were required to have measurable lesions on magnetic resonance imaging (MRI), an expected survival of at least 1 year, and a Karnofsky Performance Status score of 50 or higher. Laboratory criteria included the following serum levels: creatinine ≤ 1.5 mg/dL, uric acid ≤ 50 mg/dL, PT INR ≤ 1.5, AST ≤ 7 × the upper limit of normal, ALT ≤ 7 × the upper limit of normal, and HbA1c ≤ 6.1%. Patients with intestinal obstruction or predisposition to ileus, diabetes mellitus with HbA1c > 6.1%, or those requiring antidiabetic medication were excluded. Patients were excluded if unable to metabolize ketone bodies from fatty acids, had heart failure (NYHA class > 2), acute myocardial infarction within the past 6 months, atrial fibrillation, or infections requiring systemic treatment.

### Modified KD for Patients

**Figure 1.**
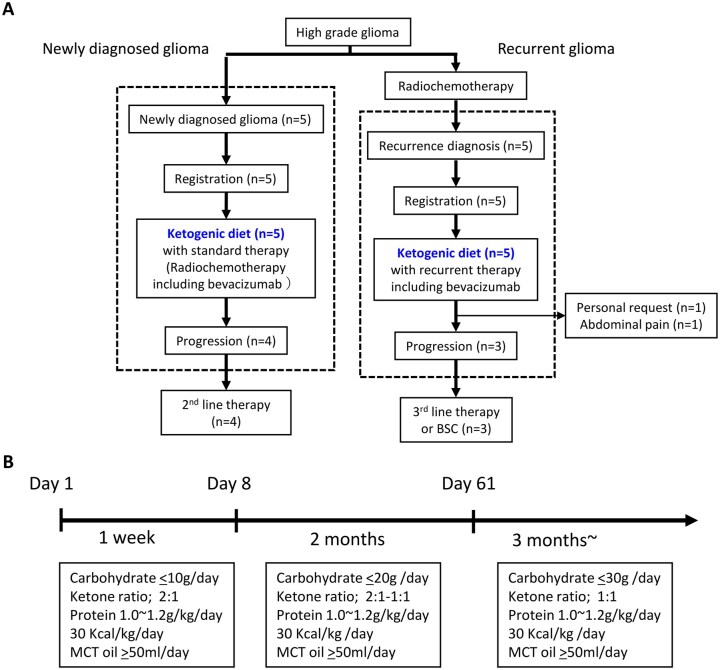
mKD regimen for patients. (A) Flow chart of the study. (B) Modified ketogenic diet (mKD) protocol for patients with high-grade glioma. Total caloric intake was set at 30-40 kcal/kg/day according to each patient’s actual body weight. The ketone ratio was calculated as lipid/(protein + carbohydrate). During the first week, carbohydrate intake was restricted to 10 g/day, with fat and protein adjusted to achieve a ketone ratio of 2:1. From the second week, carbohydrate intake was restricted to 20 g/day for 2 months. From the third month onward, carbohydrate intake was limited to 30 g/day or less. To supplement caloric intake, medium-chain triglyceride (MCT) oil (approximately 50-80 g/day) was administered. BSC, best supportive care.

Based on safety and feasibility considerations, the mKD regimen was developed by Hagihara et al. (Figure 1),^15^ which was implemented in this study. During the first week of intervention, carbohydrate intake was restricted to 10 g/day, and fat and protein amounts were adjusted to achieve a ketone ratio of 2:1 (fat/[protein + carbohydrate]). Total caloric intake was set at 30-40 kcal/kg/day, calculated using each patient’s actual body weight. From the second week, carbohydrate intake was restricted to 20 g/day for 2 months. From the third month onward, carbohydrate intake was limited to 30 g/day. To ensure sufficient caloric intake, medium-chain triglyceride (MCT) oil was administered at a dose of approximately 50-80 g/day. The MCT oil was generously provided by The Nisshin OilliO Group, Ltd (Tokyo, Japan). In addition, essential trace elements and vitamins were supplemented using oral nutritional preparations.

After providing informed consent, patients initiated the mKD under the supervision of registered dietitians from the Department of Nutrition, Kobe University Hospital. For inpatients, nutritional counseling was conducted at enrollment, before initiation of the mKD, and prior to discharge. For outpatients, counseling was provided at enrollment and during monthly follow-up visits. When necessary, commercially available mKD powders, such as ketone-formula (Meiji Co., Ltd, Tokyo, Japan) or KetoCal 4:1 (Nutricia North America, Inc., White Plains, New York, United States), were added to the diet. The Ketone–Formula was generously provided by Meiji Co., Ltd For newly diagnosed glioma patients, the KD was generally started during hospitalization at the time of initiation of chemoradiotherapy. For recurrent cases, the KD was initiated at home after confirmation of recurrence, followed by informed consent and nutritional counseling. Combined BEV was administered at 10 mg/kg every 2 weeks for hospitalized patients with newly diagnosed glioma, and at 15 mg/kg every 4 weeks for outpatients after discharge. BEV treatment was continued throughout the entire duration of the KD therapy.

### Evaluation and Assessments

Patients were monitored for body weight, vital signs, subjective and objective symptoms, and adverse events. In hospitalized patients, dietary adherence was assessed by physicians, and weekly blood tests were performed to measure blood glucose, complete blood count, serum ketone bodies, and urinary ketone bodies. In outpatients, adherence to the dietary regimen was evaluated at each clinic visit through review of dietary intake records by physicians or registered dietitians, and blood and urine tests were conducted. Body composition was evaluated using the InBody 720 device (Biospace Co. Ltd, Seoul, South Korea). Parameters measured included body weight, body mass index (BMI), skeletal muscle mass (SMM), skeletal muscle index (SMI), SMM, soft lean mass (SLM), body fat mass (BFM), and percent body fat (PBF), as needed. Imaging assessments were conducted every 2 months using MRI, and treatment response was determined according to the Response Assessment in Neuro-Oncology (RANO) criteria. The study intervention was discontinued under the following conditions: (1) voluntary withdrawal or revocation of consent by the patient; (2) resolution of the primary disease with no further treatment required; (3) disease progression making continued participation inappropriate; and (4) adverse events preventing continuation of the study.

### Statistical Analysis

Treatment response was assessed according to the RANO criteria. OS and PFS were analyzed using the Kaplan–Meier method and compared with the log-rank test. For time-to-event analyses, time zero was defined as the initiation date of the mKD, with events defined as the date of death for OS and the date of first progression for PFS.

## Results

### Patient Characteristics

Ten patients were enrolled in the study. Patient characteristics are summarized in Table 1. Five patients had newly diagnosed glioma, and 5 had recurrent glioma. The mean age was 39 years (range, 22-64), and the cohort included 7 males and 3 females. Histological diagnoses included 1 case of astrocytoma, 4 cases of glioblastoma, 2 cases of DMG, 2 cases of high-grade glioma, and 1 case of oligodendroglioma grade 3. During mKD therapy, newly diagnosed patients received radiotherapy combined with TMZ and BEV, while recurrent cases were treated with BEV and/or TMZ.

**Table 1.** Patients’ characteristics and blood test data

No.	Age	Sex	Pathology	Newly/ Rec.	IDH mut.	MGMT methyl.	KPS	EOR at initial surgery	Conbination Therapy	Duration of mKDT (days)	Reason for discontinuation	Serum total ketone (µmol/L) (mean)	Serum 3HB (µmol/L) (mean)	Blood glucose (mg/dL) (mean/ baseline)	GKI (minimum /mean)	T-Chol (mg/L) (mean/ baseline)	LDL-Chol (mg/L) (mean/ baseline)	TG (mg/L) (mean/ baseline)	TP (g/dL) (mean/ baseline)	Alb (g/dL) (mean/ baseline)	Body weight (Kg) (mean/ baseline)
1	22	M	High grade glioma, NOS	Newly	-	n.e.	60	Biopsy	RT(60Gy), TMZ·Bev	1954	-	2603±1345	1688±965	87 / 88	1.0 / 6.3	144 / 117	82 / 76	65 / 48	6.7 / 6.7	4.0 / 4.1	42.6 / 46.8
2	31	F	Glioblastoma, NF1-mutant	Newly	-	-	70	GTR	RT(60Gy), TMZ·Bev, TTF, reoperatrion	781	Disease progression	2258±995	2727±827	80 / 86	0.8 / 3.2	241 / 244	151 / 175	78 / 116	7.1 / 7.1	4.1 / 3.8	49.4 / 53.7
3	25	M	Diffuse midline glioma	Newly	-	n.e.	90	Biopsy	RT(60Gy), TMZ·Bev	244	Disease progression	2831±1003	2169±831	85 / 80	1.3 / 2.7	270 / 188	195 / 136	80 / 62	7.1 / 7.1	4.6 / 4.4	62.9 / 68.0
4	64	M	Glioblastoma	Newly	-	n.e.	80	PR	RT(60Gy), TMZ·Bev	190	Disease progression	1976±764	1415±554	93 / 89	1.3 / 5.5	201 / 210	128 / 139	78 / 55	6.0 / 5.6	4.0 / 3.6	67.3 / 69.0
5	30	M	High grade glioma, NF1-mutant	Newly	-	-	70	PR	RT(60Gy), TMZ·Bev	428	Disease progression	908±885	645±668	87 / 106	1.8 / 13.3	212 / 143	130 / 72	89 / 74	6.8 / 7.2	4.4 / 4.1	70.0 / 69.4
6	45	M	Astrocytoma, IDH-mutant	Rec.	+	+	80	Biopsy	TMZ·Bev	181	personal request	2089±1191	1435±875	101 / 101	1.8 / 9.1	210 / 231	149 / 172	140 / 144	7.6 / 7.4	4.5 / 4.3	72.8 / 81.6
7	25	M	Diffuse midline glioma	Rec.	-	n.e.	60	PR	TMZ·Bev	74	Disease progression	2619±1214	1731±943	79 / 85	1.3 / 3.2	211 / 238	141 / 190	120 / 151	7.1 / 7.2	4.4 / 4.4	62.0 / 65.0
8	52	F	Oligodendroglioma, grade 3	Rec.	+	n.e.	90	GTR	TMZ·Bev	63	AE (abdominal pain)	2633±1608	1889±1172	73 / 80	1.5 / 3.0	292 / 248	186 / 159	-	5.9 / 5.6	3.9 / 3.6	38.6 / 39.2
9	40	F	Glioblastoma	Rec.	-	n.e.	80	PR	TMZ·Bev	112	Disease progression	2659±1718	2035±1362	87 / 93	1.2 / 5.3	340 / 281	240 / 178	102 / 115	7.2 / 7.4	4.5 / 4.4	58.4 / 66.2
10	56	M	Glioblastoma	Rec.	-	n.e.	80	PR	TMZ·Bev	179	Disease progression	1069±471	959±274	92 / 91	2.9 / 6.2	207 / 187	129 / 118	59/ 116	6.3 / 6.4	4.2 / 4.2	72.0 / 73.0

Abbreviations: M, male; F, female; NOS, not otherwise specified; IDH, isocitrate dehydrogenase; MGMT, O-6-methylguanine-DNA methyltransferase; KPS, Karnofsky performanc status; EOR, extent of resection; RT, radiation therapy; TMZ, temozolomide; Bev, bevacizumab; KDT, ketogenic diet therapy; TTF, tumor treating fields; 3HB, 3-hydroxybutyrate; GKI , glucose-ketone index; T-Chol, total cholesterol; LDL-Chol, low-density lipoprotein cholesterol; TG, triglyceride; TP, total protein; Alb, albumin; n.e., not evaluated

### Adherence and Tolerability

The duration of mKD varied across patients, ranging from 63 days (2.1 months) to 1954 days (5.4 years) (Table 1). The median duration was 185 days (6.2 months), and the mean duration was 421 days (14.0 months). The duration tended to be longer in newly diagnosed gliomas compared with recurrent cases (*P* = .1). The mean duration of mKD in newly diagnosed glioma was 719 days (24 months), whereas in recurrent cases it was 121 days (4 months). Reasons for discontinuation of mKD were varied: 7 patients discontinued due to progressive disease, 1 withdrew from the study owing to personal reasons, and 1 discontinued due to abdominal pain associated with MCT oil (Table 1). Given that 60% of patients continued the KD for at least 6 months and that all newly diagnosed cases maintained the diet beyond this period, we concluded that the feasibility of this mKD was achieved.

### Body Composition and Blood Test after mKD

**Figure 2.**
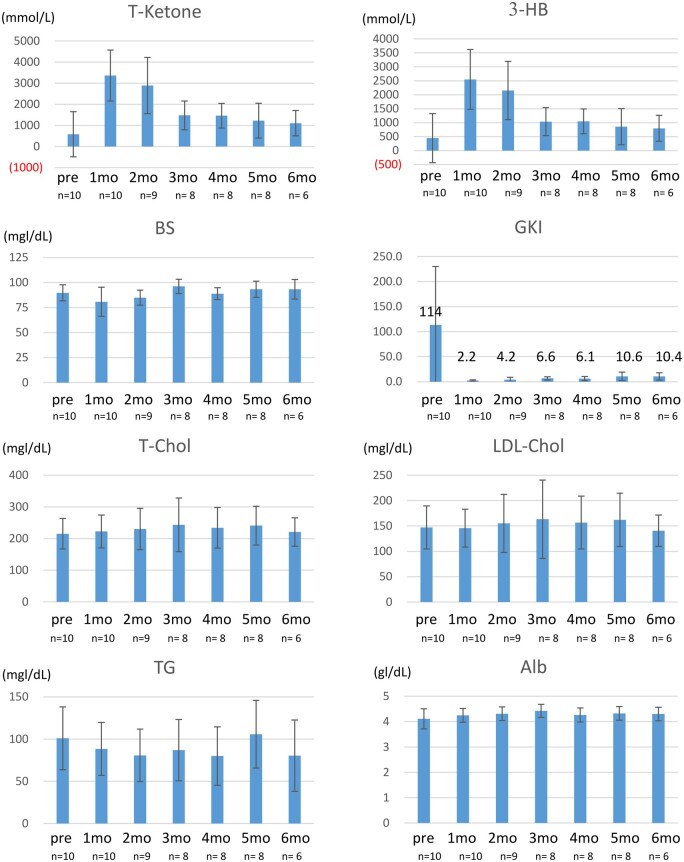
Blood data of patients after the mKD. Bar graphs represent the mean value of T-Chol, 3-HB, BS, GKI, T-Chol, LDL-C, TG, and Alb levels during the first 6 months. Notably, T-ketone and 3-HB levels were significantly elevated at 1 month, while GKI values decreased to approximately 2-10. Abbreviations: T-ketone, total ketone; 3-HB, β-hydroxybutyrate; BS, blood sugar; GKI, glucose-ketone index; T-Chol, total cholesterol; LDL-C, low density lipoprotein cholesterol; TG, triglyceride; Alb, albumin.

Nine out of ten patients exhibited mild weight loss (Table 1, Supplementary Figure S1). No statistically significant changes were observed in BMI, SMI, SLM, BFM, or PBF (Supplementary Figure S1). The mean serum total ketone, 3-hydroxybutyrate (3-HB), and blood glucose levels during mKD therapy were 2324 µmol/L, 1675 µmol/L, and 82 mg/dL, respectively (Table 1 and Figure 2). 1 month after initiation of the mKD, mean total ketone and 3-HB levels increased to 3365 µmol/L and 2549 µmol/L, respectively (Figure 2). The minimum glucose-ketone index (GKI) value was observed within 1-2 months after initiation of KD, with a mean of 1.49 (range, 0.8-2.9). However, the average GKI during the KD period was 5.8 (range, 3.19-13.3). Ketone levels tended to decline in all patients following discharge. No patients experienced hypoglycemia or ketoacidosis. Baseline and mean values during mKD therapy for total cholesterol (T-Chol), LDL-cholesterol (LDL-C), triglycerides, total protein, and albumin levels are presented in Table 1 and Figure 2. No significant changes were observed with mKD therapy.

### Safety and Adverse Events

All 10 patients met the primary safety endpoint defined for this trial, with no severe adverse events reported. Adherence to mKD was high, with all patients (100%) maintaining nutritional ketosis (≥0.3 mM) for >50% of study days (Table 1). Adverse events were observed in 5 patients (Table 2), including abdominal pain, diarrhea, appendicitis, skin disorder, nausea/vomiting, seizure, and taste disturbance. Skin disorder and taste disturbance improved with vitamin supplementation, while diarrhea and nausea/vomiting resolved after modifying the method of MCT oil intake. Regarding appendicitis, it was mild and considered unlikely to be caused by the KD. In the patient with abdominal pain, the symptom was attributed to MCT oil, and mKD was discontinued after 2 months.

**Table 2.** Treatment response and prognosis

No.	Pathology	Newly/ Rec.	Adverse events	Response rate	Recurrence	Recurrent pattern	PFS after KDT (months)	OS after KDT (months)	OS after initial therapy (months)	Outcome
1	High grade glioma, NOS	Newly	Seizure	PR	−	−	65	65	68	Alive
2	Glioblastoma, NF1-mutant	Newly	Epidermal peeling, Taste disorder, Appendicitis	CR	+	Local	22	31	33	Dead
3	Diffuse midline glioma	Newly	−	PR	+	Dissemination	6	11	12	Dead
4	Glioblastoma	Newly	−	PD	+	Dissemination	5	7	8	Dead
5	High grade glioma, NF1-mutant	Newly	−	CR	+	Distant	13	18	19	Alive
6	Astrocytoma, IDH-mutant	Rec.	Diarrhea	SD	+	Local	36	40	61	Alive
7	Diffuse midline glioma	Rec.	−	PD	+	Local	2	4	31	Dead
8	Oligodendroglioma, grade 3	Rec.	Abdominal pain	SD	−	−	20	20	59	Alive
9	Glioblastoma	Rec.	−	SD	+	Local	4	7	14	Dead
10	Glioblastoma	Rec.	Nausea, Vomiting	PR	+	Distant	6	10	41	Alive

Abbreviations: CR, complete response; PR, partial response; SD, stable disease; PD, progressive disease; PFS, progression free survival; OS, overall survival; KDT, ketogenic diet therapy.

### Treatment Response and Survival

**Figure 3.**
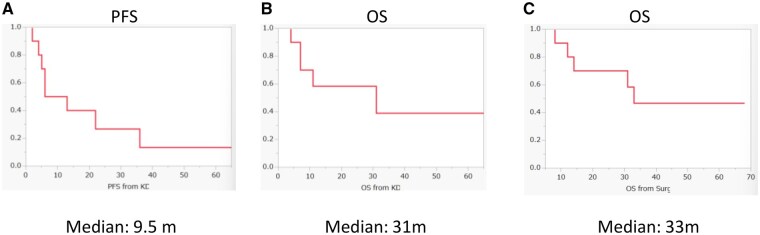
Patient survival curves. (A) Progression-free survival (PFS) curve from initiation of mKD in all patients (*n* = 10). Median PFS was 9.5 months. (B) Overall survival (OS) curve from initiation of mKD in all patients. Median OS was 31 months. (C) Overall survival curve from initial surgery in all patients. Median OS was 33 months.

At the time of analysis, 5 patients were alive and 5 had died. Regarding treatment response, 1 patient achieved a complete response, 4 had partial responses, 3 exhibited stable disease, and 2 experienced progressive disease (Table 2). The objective response rate was 50%. One patient (Patient #1) has remained on mKD for more than 5 years and continues the diet with no evidence of tumor recurrence. Kaplan–Meier curves for PFS and OS are shown in Figure 3. From the initiation of mKD (Day 1), the median PFS was 9.5 months (Figure 3A), and the median OS was 31 months (Figure 3B). From the date of diagnosis, the median OS was 33 months (Figure 3C). In the subset of 5 patients with newly diagnosed malignant glioma, the median OS was 18 months, and the median progression-free survival was 13 months. No significant differences were observed between newly diagnosed group and recurrent group (Supplementary Figure S2).

### Case Illustrations of Newly Diagnosed Patients

#### Patient #1

A 22-year-old man initially presented to a local hospital with visual disturbance. MRI revealed a bilateral thalamic tumor (Supplementary Figure S3A-a). He underwent craniotomy for tissue biopsy, and histopathological examination confirmed a diagnosis of high-grade glioma NOS. Following surgery, the patient was referred to our institution for further treatment. Postoperatively, he received standard radiotherapy (2 Gy × 30 fractions; total dose, 60 Gy) combined with chemotherapy using TMZ and Bev. After completing initial chemoradiotherapy, mKD was initiated concurrently with maintenance TMZ and Bev in the outpatient setting. Serum ketone and 3-HB levels increased to over 5000 µmol/L and 2500 µmol/L, respectively, and the GKI was maintained at approximately 2 (Supplementary Figure S3B). 2 months after initiation of mKD, new contrast-enhancing lesions were observed in the right basal ganglia and corpus callosum (Supplementary Figure S3A-b). Nevertheless, mKD and chemotherapy were continued. Subsequent imaging showed gradual tumor regression, and no recurrence has been observed for 5 years (Supplementary Figure S3A-c, d). The patient remains on mKD and continues to receive maintenance Bev every 3 months in the outpatient clinic.

#### Patient #2

**Figure 4.**
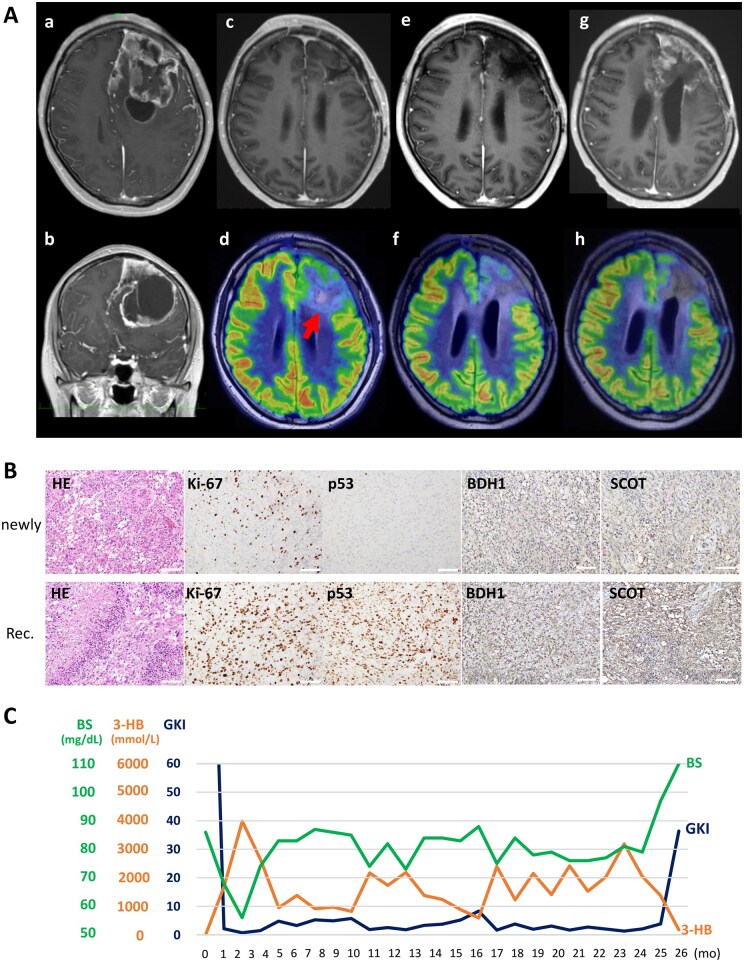
Illustration of Patient #2. (A) Serial neuroimaging of a 32-year-old woman with neurofibromatosis type 2 (NF-2) and left frontal glioblastoma. (a, b) Pre-treatment gadolinium-enhanced MRI showing a large contrast-enhancing tumor. (c, d) Postoperative Gd-enhanced MRI and FDG-PET demonstrating residual tumor. (e, f) MRI and FDG-PET 1 year after treatment initiation, showing tumor regression. (g, h) MRI and FDG-PET 26 months after treatment initiation, revealing local recurrence. The enhancing lesion showed minimal uptake on FDG-PET. (B) Histopathological findings of the tumor. Upper panels: primary tumor. Lower panels: recurrent lesion, which demonstrated extensive necrosis, a high Ki-67 labeling index, and strong immunopositivity for p53. Expression of ketone-metabolizing enzymes BDH1 and SCOT was increased. HE, BDH1, SCOT: Original magnification x200, Scale Bar: 100 μM. Ki-67, p53: Original magnification x400, Scale Bar: 50 μM. (C) Longitudinal monitoring of serum 3-HB (orange), blood glucose (green), and GKI (dark blue) during mKD therapy. Sustained ketosis and consistently low GKI values were achieved throughout the treatment course.

A 32-year-old woman with a history of neurofibromatosis type 2 (NF-2) was admitted with headache and visual disturbance. MRI revealed a large gadolinium-enhancing tumor with a cystic component in the left frontal lobe (Figure 4A-a, b). The tumor was grossly resected without any postoperative neurological deficits, including motor weakness or aphasia. Histopathological examination confirmed glioblastoma (Figure 4B, upper). Following surgery, the patient underwent standard radiotherapy (60 Gy) with concomitant TMZ and Bev. In addition, mKD was initiated at the start of radiotherapy. After completing initial radiochemotherapy, the patient was discharged and continued mKD under the guidance of a registered dietitian, along with maintenance TMZ and Bev in the outpatient setting. Throughout therapy, serum 3-HB levels remained consistently above 1,000 µmol/L, and GKI was maintained between 2 and 3 (Figure 4C). The residual tumor gradually regressed (Figure 4A-e, f); however, 26 months after initiation of mKD, recurrence occurred in the left frontal lobe (Figure 4A-g, h). A second craniotomy was performed, and subtotal resection of the recurrent lesion was achieved. Histological analysis revealed extensive necrosis, a high Ki-67 labeling index, and strong p53 immunopositivity. Notably, ketone-metabolizing enzymes, including BDH1 and SCOT, were highly expressed in tumor cells (Figure 4B, lower). Despite multimodal treatment, the patient died owing to tumor progression 33 months after therapy initiation.

#### Patient #3

A 25-year-old man presented with headache and was admitted to a hospital, where MRI and FDG-PET revealed a left thalamic tumor (Supplementary Figure S4A-a-c). Stereotactic biopsy confirmed a diagnosis of DMG with the H3K27M mutation. He underwent concurrent chemoradiotherapy with TMZ and Bev, along with mKD therapy initiated during hospitalization. Serum 3-HB levels increased to 3,000 µmol/L without any associated adverse events (Supplementary Figure S4B). The 6-week initial treatment course was completed without interruption, and maintenance therapy was continued in the outpatient setting. The mKD was maintained under the supervision of a registered dietitian, and sustained nutritional ketosis was achieved. However, 6 months after the initiation of mKD, the patient developed severe headache, and spinal dissemination was detected on MRI despite a reduction in the size of the thalamic tumor (Supplementary Figure S4A-d-f). Consequently, both mKD and chemotherapy were discontinued, and treatment was shifted to best supportive care. The patient ultimately died owing to tumor progression, with an OS of 11 months from mKD initiation.

#### Patient #4

A 64-year-old man presented with a headache and was admitted to a local hospital, where he underwent surgical resection of a right frontal lobe tumor. Histopathological examination confirmed glioblastoma. He was subsequently referred to our institution, where postoperative treatment with radiotherapy (60 Gy) and concomitant chemotherapy with TMZ and Bev was initiated (Supplementary Figure S5A-a, b). Simultaneously, the patient initiated mKD. Serum total ketone and 3-HB levels promptly increased to 3,800 µmol/L and 2,700 µmol/L, respectively, and a favorable GKI was maintained during the initial treatment course (Supplementary Figure S5B). After completion of chemoradiotherapy, contrast-enhancing lesions on MRI had markedly diminished (Supplementary Figure S5A-c). However, 5 months after the initiation of therapy, the patient developed back and muscle pain. MRI revealed tumor recurrence (Supplementary Figure S5A-d), and spinal MRI demonstrated linear contrast enhancement along the surface of the spinal cord (Supplementary Figure S5A-e). Cerebrospinal fluid analysis confirmed malignant cells, suggestive of spinal dissemination (Supplementary Figure S5A-f). Given disease progression, all active treatments, including mKD, were discontinued, and the patient transitioned to best supportive care. He died owing to disease progression 7 months after initiation of mKD therapy.

#### Patient #5

A 30-year-old man presented with headache and mild disturbance of consciousness and was admitted to our hospital. MRI revealed a left thalamic tumor associated with obstructive hydrocephalus (Supplementary Figure S6A-a). Emergent ventricular drainage was performed, followed by tumor resection via the interhemispheric transventricular approach after 1 week (Supplementary Figure S6A-b, f). Postoperatively, the patient received standard radiochemotherapy with TMZ and Bev, mKD therapy was initiated concurrently. Serum total ketone and 3-HB levels rapidly increased to over 5,000 µmol/L and 4,000 µmol/L, respectively (Supplementary Figure S6B). No adverse events were observed during the initial treatment phase. In the outpatient setting, ketone and 3-HB levels stabilized at approximately 300-500 µmol/L. Both the mKD and maintenance chemotherapy (TMZ + Bev) were continued. Ten months after initiation of treatment, the tumor shrank and disappeared (Supplementary Figure S6A-c, g). However, 3 months later, new lesions were detected in the left cerebellar hemisphere, which showed increased uptake on FDG-PET imaging (Supplementary Figure S6A-d, h). At that point, mKD therapy was discontinued, and the cerebellar lesions were treated with radiotherapy. The patient continues to receive maintenance TMZ and Bev.

## Discussion

Based on our previous findings, we conducted a clinical study to evaluate the safety and feasibility of incorporating a KD into the standard treatment regimen, including an angiogenesis inhibitor, for patients with malignant glioma. In this study, we employed the mKD developed by Hagihara et al. which emphasizes carbohydrate restriction rather than the traditional ketogenic ratio. All 5 newly diagnosed patients received concurrent chemoradiotherapy, exhibited a marked reduction in GKI to <2 during the 1st month of inpatient treatment, and were able to continue the mKD for more than 6 months. Two of these patients demonstrated reduced glucose uptake in residual lesions on FDG-PET imaging, and the median OS in the newly diagnosed group was 18 months, suggesting a potential therapeutic benefit. Compared with recurrent cases, newly diagnosed patients adhered to the diet for a significantly longer duration and maintained lower GKI levels, indicating that the mKD may be more feasible and sustainable in this population. The prolonged survival of one glioblastoma patient (Patient #2), who lived for 33 months, further raises the possibility that the combination of KD and BEV contributed to this favorable outcome. In the recurrent cohort, long-term survival was observed in patients with *IDH*-mutant tumors. Given the distinct metabolic phenotype characteristic of *IDH*-mutant gliomas, it is plausible that these tumors were particularly responsive to the metabolic alterations induced by KD and BEV.

### Glucose Metabolism in Glioma Cells

Glioma cells exhibit a pronounced reliance on aerobic glycolysis (the Warburg effect), generating ATP as well as metabolic intermediates that sustain cellular proliferation and redox homeostasis, even under hypoxic conditions. This metabolic reprogramming is largely driven by constitutive activation of the PI3K/Akt/mTOR signaling cascade, frequently upregulated via receptor tyrosine kinases such as IGFR, EGFR, and PDGFR.^16^^,^^17^ Activation of this pathway promotes HIF-1–mediated upregulation of glucose transporters, thereby facilitating increased glucose uptake and a metabolic shift toward glycolysis.^18^

In contrast to normal glial and neuronal cells, glioma cells display markedly reduced expression of key ketolytic enzymes, including BDH1 and SCOT, along with mitochondrial dysfunction, resulting in limited capacity to utilize ketone bodies as an alternative energy substrate.^6^^,^^19^ Consequently, glucose deprivation induces apoptosis in glioblastoma cells, while normal glia remains resistant.^20–22^

Preclinical models have demonstrated that suppression of glucose availability combined with elevation of ketone bodies reprograms tumor metabolism and suppresses tumor growth, findings consistent with clinical observations that hyperglycemia correlates with poor prognosis in glioblastoma.^6^^,^^23–27^ Carbohydrate restriction may further enhance tumor sensitivity to chemotherapy and radiotherapy. In addition, the major ketone body 3-HB possesses antioxidant and neuroprotective properties, which may attenuate treatment-related toxicity and mitigate therapy-associated aging.^28–30^

### Antitumor Effect of KD in Combination with an Anti-angiogenic Agent in vivo

In a brain tumor mouse model, KD enhances radiosensitivity and significantly prolong survival.^31^ In our previous murine xenograft model, the combination of KD and the anti-angiogenic agent BEV induced marked alterations in intratumoral metabolites.^6^ Although KD alone increased intratumoral amino acid concentrations, reflecting enhanced amino acid metabolism, concomitant administration of BEV markedly reduced these levels. This suppression of amino acid metabolism was associated with cell cycle inhibition and attenuation of tumor growth. These findings suggest that the therapeutic efficacy of KD alone may be limited, whereas its combination with anti-angiogenic therapy may exert synergistic antitumor effects. Based on this rationale, the present study evaluated the feasibility of implementing KD in conjunction with standard therapy, with all patients receiving BEV.

### Clinical Study of KD Therapy in Glioma

In recent years, an increasing number of studies have investigated KD therapy in patients with glioma. Schwartz et al. reported 2 cases of recurrent glioblastoma treated with KD, which was safe and feasible, although PFS was limited to 4 and 12 weeks, respectively.^10^ Elsakka et al. described a glioblastoma patient who received KD in combination with metformin, chloroquine, and hyperbaric oxygen therapy in addition to standard treatment, achieving disease control for 2 years.^32^ van der Louw et al. studied nine patients with newly diagnosed glioblastoma who received KD alongside standard therapy, reporting OS of 9.9-19.0 months.^33^ Woodhouse et al. evaluated 29 patients with glioma treated with KD and radiochemotherapy and demonstrated enhanced radiosensitivity.^34^ Similarly, Panhans et al. treated 12 patients with KD and standard therapy, observing radiographic tumor shrinkage and improved edema.^35^ Klein et al. administered a strict ketogenic regimen (≤10 g/day of carbohydrates, 1600 kcal/day, ketogenic ratio 4:1) to a patient with newly diagnosed glioblastoma, reporting a survival of 11-29.2 months.^36^ Martin-McGill et al. conducted a single-center, randomized pilot study involving 12 patients with newly diagnosed glioblastoma, randomized 1:1 to receive either a modified KD or a MCT KD.^37^ However, only 4 of the 12 patients completed the prescribed 3-month intervention, highlighting the importance of dietary composition and patient motivation for adherence. Most recently, Amaral et al. conducted a phase I clinical trial evaluating KD therapy in 17 patients with glioblastoma.^14^ The study demonstrated high adherence, with median PFS and OS of 12.9 months and 29.4 months, respectively, from KD initiation. Importantly, quality of life, symptom control, and cognitive function were maintained or improved throughout the intervention. Although these reports consist largely of case series with heterogeneous standard treatments, variable diet composition, and differences in initiation timing and duration, the collective findings suggest that KD can be safely implemented in glioma patients without serious adverse events when used alongside standard radiochemotherapy.

Duraj and colleagues emphasized that maintaining a GKI of ≤2, and ideally ≤1, is essential for achieving therapeutic ketosis and exerting antitumor efficacy.^38^ They further highlighted that while KD effectively suppresses glycolysis, it does not adequately inhibit glutaminolysis. Accordingly, they proposed combination strategies guided by the “press–pulse principle,” in which KD provides the sustained metabolic pressure (press), while glutamine-targeted inhibitors or cytotoxic agents are administered intermittently and dose-escalated as “pulses.” This paradigm aims to selectively exploit metabolic vulnerabilities of glioma cells while minimizing collateral toxicity to normal tissue.

In our clinical trial, however, the mean GKI during the KD period was 5.8. When the duration of GKI ≤2 was examined, the mean across the 10 patients was 2.9 months. These findings suggest that more stringent dietary regulation may be required to achieve sustained therapeutic ketosis and maximize potential antitumor benefits. Accordingly, maintaining a GKI below 2 necessitates strategies that both enhance β-hydroxybutyrate (3HB) production and lower blood glucose levels.

### Clinical Study of KD Combined with an Angiogenesis Inhibitor in Glioma

We previously demonstrated in a murine brain tumor xenograft model that combining KD with the angiogenesis inhibitor BEV induced profound alterations in the intratumoral metabolite profile.^6^ KD alone increased concentrations of various amino acids within the tumor, whereas the addition of BEV markedly reduced these levels. In particular, glutamic acid, aspartic acid, tryptophan, phenylalanine, and serine levels were significantly decreased with the combined treatment.^6^ BEV reduces tumor vascularization and induces ischemia by diminishing intratumoral blood flow^39^^,^^40^; hence, its combination with KD may further restrict nutrient availability and create a state of metabolic deprivation within the tumor microenvironment. The combination of a KD and BEV may exert greater therapeutic efficacy in tumors with abundant blood flow and strong dependence on glucose metabolism. Clinical data on the combined use of KD and anti-angiogenic therapy in glioma patients remain limited. Rieger et al. reported on 7 patients with recurrent glioblastoma treated with KD and BEV; all patients exhibited objective responses, with a median PFS of 20.1 weeks under BEV treatment.^11^ Klein et al. described a single case of recurrent glioblastoma treated with the same combination, in which the patient experienced marked symptom improvement, tumor regression, and resolution of cerebral edema, ultimately surviving for 20 months after KD initiation.^36^ Given the paucity of clinical evidence, further studies with larger cohorts are warranted to evaluate the therapeutic potential and mechanistic synergy of KD combined with angiogenesis inhibitors in glioma management. Notably, 2 large-scale phase III clinical trials assessing BEV in combination with standard radiotherapy and TMZ chemotherapy failed to show a significant improvement in OS.^41^^,^^42^ However, integration with KD may enhance therapeutic efficacy and has the potential to extend OS. There are 2 cases of complete response, all of which have NF1 nutation.

### Adverse Events of mKD

Various adverse events associated with KD have been reported.^43^ In the early stages of treatment, common adverse events include fatigue, headache, dizziness, nausea, and gastrointestinal disturbances such as constipation and diarrhea. These symptoms are typically attributed to the metabolic shift induced by the abrupt reduction in carbohydrate intake and often resolve within several days to weeks. Abdominal symptoms are also frequently observed due to MCT oil consumption. A high-fat, low-fiber diet has been linked to reduced gut microbial diversity. The high fat content of KD may lead to hyperlipidemia, including elevated LDL cholesterol and T-Chol levels. Long-term adherence to a high-fat diet has also been associated with increased risk of hepatic fat accumulation.^43^ Other reported complications include deficiencies in vitamins and minerals and decreased bone density.^44^ In pediatric populations, KD has been associated with reduced bone mineral density, increased fracture risk, and growth retardation. Moreover, the risk of nephrolithiasis (kidney stones) is elevated, with one meta-analysis reporting an incidence of 5.9% over 4 years, which is approximately 7 to 8 times higher than the annual incidence in the general adult population.^45^

In the present study, abdominal symptoms such as diarrhea and nausea/vomiting were observed, along with skin abnormalities and taste disturbances attributable to vitamin deficiency. All these adverse events were mild and resolved promptly. Additional events included one case of abdominal pain and one case of appendicitis. The abdominal pain was attributed to MCT oil intolerance and led to discontinuation of the mKD in patient 7. Given the paucity of reported cases, a causal association between appendicitis and the KD appears unlikely. Nevertheless, continuation of mKD therapy remained feasible, supporting its overall favorable tolerability and practicality.

### Limitations

This study has several limitations. First, the small sample size limits the generalizability of the findings. Second, the histopathological subtypes of glioma varied among patients, and both newly diagnosed and recurrent cases were included. Third, concomitant treatments administered alongside mKD were not standardized and differed among individual cases. However, in patients with newly diagnosed glioma, standard initial therapy—comprising radiotherapy, concomitant TMZ, and BEV—was consistently applied, and mKD was successfully maintained throughout the initial treatment phase in all 5 cases. Another limitation is that during outpatient maintenance therapy, only monthly blood tests were performed, whereas the more detailed monitoring possible during hospitalization could not be replicated. In addition, in our KD protocol, the mean GKI exceeded 6 after the 3-month mark. As therapeutic KD generally recommend maintaining a GKI of ideally ≤3, it may be necessary to reassess the degree of carbohydrate restriction and the intake of MCT oil beyond this period. However, the optimal GKI threshold when the KD is combined with BEV remains unknown, and further investigation is warranted.

Despite these limitations, all patients in this study successfully received combined treatment with mKD and BEV, with minimal adverse events observed. Therefore, larger studies are warranted to further evaluate the safety and efficacy of this therapeutic approach.

### Future Directions

KD therapy induces systemic metabolic reprogramming toward a state of nutrient deprivation that is inherently unfavorable for tumor growth. However, KD monotherapy may promote adaptive metabolic remodeling within tumor cells, ultimately leading to therapeutic resistance. To overcome this, comprehensive and combinatorial treatment strategies are needed. Antidiabetic agents are of particular interest because of their ability to suppress blood glucose and insulin signaling, thereby amplifying the metabolic impact of KD. Metformin, at standard therapeutic doses, mildly inhibits hepatic gluconeogenesis and enhances insulin sensitivity, resulting in improved glycemic control. Furthermore, metformin exerts immunomodulatory effects within the tumor microenvironment. Given its established safety profile, multiple clinical trials are currently evaluating metformin as an adjuvant to standard-of-care (SOC) cancer therapies. Sodium-glucose cotransporter 2 (SGLT2) inhibitors—such as dapagliflozin, empagliflozin, and canagliflozin—represent another class of glucose-lowering agents that further attenuate insulin signaling.^46^ Recent preclinical studies have demonstrated marked synergistic anti-tumor effects when SGLT2 inhibitors are combined with KD, underscoring their therapeutic potential.^46^ However, inhibition of glycolysis alone may be insufficient, as cancer cells can compensate by utilizing glutamine—another principal fermentable substrate—to sustain survival and proliferation. Furthermore, our preclinical studies demonstrated that KD therapy alone increased intratumoral glutamine concentrations and enhanced glutamine metabolism, suggesting a potential mechanism of therapeutic resistance. Therapeutic strategies targeting glutamine metabolism are therefore important. Novel glutamine antagonists, including prodrugs of 6-diazo-5-oxo-L-norleucine and agents such as CB-839, are currently under investigation as adjuncts to SOC regimens.

Collectively, these findings support the integration of mKD therapy into conventional tumor treatment frameworks. Future development of pharmacologic agents acting synergistically with mKD holds considerable promise for enhancing therapeutic efficacy and improving clinical outcomes.

## Conclusions

mKD was demonstrated to be a safe and feasible adjunct to standard radiochemotherapy, including BEV, in patients with malignant glioma. Under the dietary protocol, all patients achieved a prompt elevation in serum ketone levels and reached therapeutically sufficient GKI values, without any serious adverse events. While some patients derived clinical benefit, others experienced distant recurrence or spinal dissemination. Therefore, comprehensive, large-scale clinical trials are warranted to clarify the antitumor potential and clinical utility of mKD.

## Supplementary Material

vdaf264_Supplementary_Data

## Data Availability

The datasets generated and/or analyzed during the current study are available from the corresponding author on reasonable request.
