# Supplementary material for: Ketogenic diet therapy for high-grade gliomas combined with standard treatment using an angiogenesis inhibitor: An exploratory pilot study on feasibility
Source: Neurooncol Adv. 2025 Dec 22;8(1):vdaf264. doi: 10.1093/noajnl/vdaf264 (PMC12924638; doi:10.1093/noajnl/vdaf264)
Supplement: vdaf264_Supplementary_Data [file vdaf264_supplementary_data.zip › Supplementary legend clean.docx]

**Supplementary Figure 1: Longitudinal Changes in Body Composition**

Serial InBody measurements in one patient from pretreatment to 6 months after therapy initiation. (a) Body weight, (b) Soft lean mass (SLM), (c) skeletal muscle index (SMI) (d) Body mass index (BMI), (e) Percentage of body fat (f) Body fat mass

**Supplementary Figure 2:**

Progression free survival (PFS) and overall survival (OS) curve between newly diagnosed glioma cohort and recurrent cohort. There are no significant differences.

**Supplementary Figure 3: Illustration of Patient #1**

A: Serial neuroimaging of a 22-year-old man with thalamic astrocytoma. (a) Preoperative Gd-enhanced MRI revealed bilateral thalamic tumors. (b) Two months after initiation of mKD, new contrast-enhancing lesions appeared in the right basal ganglia and corpus callosum. (c) One year after treatment initiation, tumor size was reduced. (d) Two years after treatment, further tumor regression was observed. The patient remains on mKD and continues to receive maintenance bevacizumab every 3 months in the outpatient setting. B: Longitudinal monitoring of serum 3-hydroxybutyrate (3-HB, orange), blood glucose (green), and glucose-ketone index (GKI, dark blue) during mKD therapy.

**Supplementary Figure 4: Illustration of Patient #3**

A: Serial neuroimaging of a 25-year-old man with a left thalamic diffuse midline glioma. (a): Preoperative MRI showing a left thalamic tumor; stereotactic biopsy confirmed diffuse midline glioma with an H3K27M mutation. (b) Postoperative FDG-PET demonstrating intense FDG uptake in the tumor. (c) MRI following chemoradiotherapy combined with mKD therapy, showing slight tumor regression. (d–f) Spinal MRI performed 6 months after initiation of mKD revealed spinal dissemination, despite continued regression of the primary thalamic lesion. The patient ultimately died owing to tumor progression, with overall survival of 11 months from the start of mKD. B: Longitudinal monitoring of serum 3-hydroxybutyrate (3-HB, orange), blood glucose (green), and glucose-ketone index (GKI, dark blue) during mKD therapy. Sustained ketosis and consistently low GKI values were maintained throughout the treatment.

**Supplementary Figure 5: Illustration of Patient #4**

A: Serial neuroimaging of a 64-year-old man with glioblastoma involving the right frontal lobe and basal ganglia. (a) Postoperative MRI showing residual tumor in the right frontal lobe and basal ganglia. (b) Postoperative FDG-PET demonstrating moderate FDG uptake within the tumor. (c) MRI after completion of chemoradiotherapy combined with mKD therapy, showing tumor regression. (d) MRI during the outpatient phase, 5 months after initiation of mKD, revealing tumor recurrence at the original site and along the surface of the lateral ventricle. (e) Gadolinium-enhanced spinal MRI 7 months after initiation of mKD showing spinal dissemination. (f) Cytological examination of cerebrospinal fluid (CSF) demonstrating malignant tumor cells. B: Longitudinal monitoring of serum 3-HB (orange), blood glucose (green), and GKI (dark blue) during mKD therapy. Sustained ketosis and consistently low GKI values were maintained throughout the course.

**Supplementary Figure 6: Illustration of Patient #5**

A: Serial neuroimaging of a 30-year-old man with a left thalamic high-grade glioma.

(a) Preoperative MRI showing a left thalamic tumor with obstructive hydrocephalus. (b) Postoperative MRI showing residual tumor in the left thalamus. (c) The contrast-enhancing lesion had resolved 10 months after initiation. (d) Thirteen months after treatment initiation, FLAIR imaging revealed new hyperintense lesions in the left cerebellar hemisphere. (e) Postoperative FDG-PET demonstrating high FDG uptake in the residual thalamic tumor. (f) FDG-PET three months after treatment showing markedly reduced uptake in the thalamic region.
