# Supplementary figures and images for: Ketogenic diet therapy for high-grade gliomas combined with standard treatment using an angiogenesis inhibitor: An exploratory pilot study on feasibility
Source: Neurooncol Adv. 2025 Dec 22;8(1):vdaf264. doi: 10.1093/noajnl/vdaf264 (PMC12924638; doi:10.1093/noajnl/vdaf264)

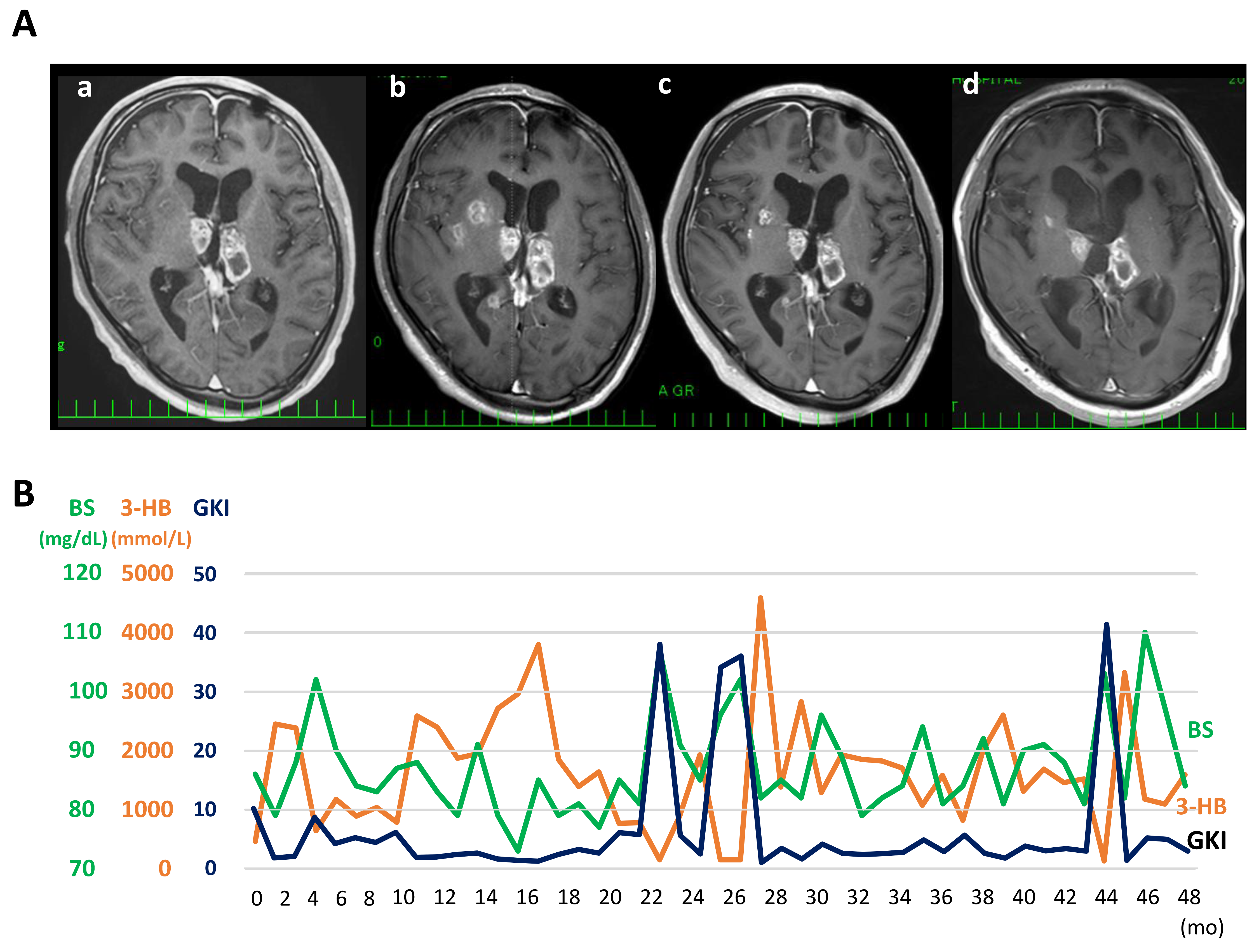

Supplement: vdaf264_Supplementary_Data [file vdaf264_supplementary_data.zip › Suppl. Figure 3.tif]

Supplemental Table


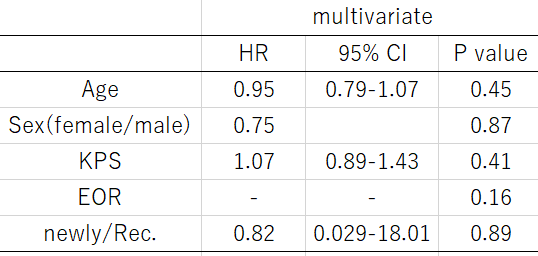


KPS: Karnofsky performance status, EOR: extent of resection

Supplement: vdaf264_Supplementary_Data [file vdaf264_supplementary_data.zip › Supplemental Table.docx]

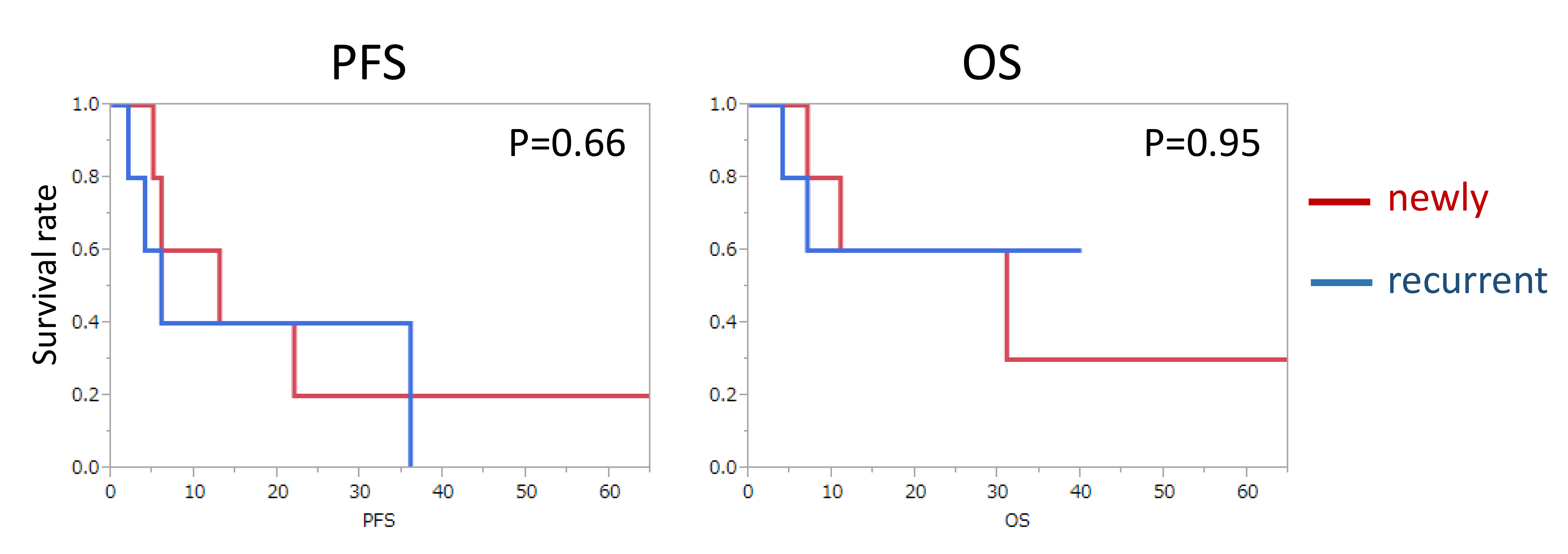

Supplement: vdaf264_Supplementary_Data [file vdaf264_supplementary_data.zip › Suppl. Figure 2.tif]
